# Inclisiran, Low-Density Lipoprotein Cholesterol and Lipoprotein (a)

**DOI:** 10.3390/ph16040577

**Published:** 2023-04-12

**Authors:** Niki Katsiki, Michal Vrablik, Maciej Banach, Ioanna Gouni-Berthold

**Affiliations:** 1 Department of Nutritional Sciences and Dietetics, International Hellenic University, 574 00 Thessaloniki, Greece; 2 School of Medicine, European University Cyprus, Nicosia 2404, Cyprus; 3 Third Department of Medicine-Department of Endocrinology and Metabolism of the First Faculty of Medicine, Charles University and General University Hospital, 121 08 Prague, Czech Republic; 4 Department of Preventive Cardiology and Lipidology, Medical University of Lodz and Polish Mother’s Memorial Hospital Research Institute, 93-338 Lodz, Poland; 5 Center for Endocrinology, Diabetes and Preventive Medicine, University of Cologne, Faculty of Medicine and University Hospital Cologne, Kerpener Str. 62, 50937 Cologne, Germany

**Keywords:** inclisiran, atherosclerotic cardiovascular disease, proprotein convertase subtilisin/kexin type 9 inhibitor, low-density lipoprotein cholesterol, lipoprotein (a)

## Abstract

Dyslipidemia treatment is of major importance in reducing the risk of atherosclerotic cardiovascular disease (ASCVD), which is still the most common cause of death worldwide. During the last decade, a novel lipid-lowering drug category has emerged, i.e., proprotein convertase subtilisin/kexin type 9 (PCSK9) inhibitors. Apart from the two available anti-PCSK9 monoclonal antibodies (alirocumab and evolocumab), other nucleic acid-based therapies that inhibit or “silence” the expression of PCSK9 are being developed. Among them, inclisiran is the first-in-class small interfering RNA (siRNA) against PCSK9 that has been approved by both the US Food and Drug Administration (FDA) and the European Medicines Agency (EMA) for the treatment of hypercholesterolemia. Importantly, inclisiran therapy may improve low-density lipoprotein cholesterol (LDL-C) target achievement by offering a prolonged and significant LDL-C-lowering effect with the administration of only two doses per year. The present narrative review discusses the **ORION/VICTORION** clinical trial program that has been designed to investigate the impact of inclisiran on atherogenic lipoproteins and major adverse cardiac events in different patient populations. The results of the completed clinical trials are presented, focusing on the effects of inclisiran on LDL-C and lipoprotein (a) (Lp(a)) levels as well as on other lipid parameters such as apolipoprotein B and non-high-density lipoprotein cholesterol (non-HDL-C). Ongoing clinical trials with inclisiran are also discussed.

## 1. Introduction

Atherosclerotic cardiovascular (CV) disease (ASCVD) represents a major cause of death worldwide [1]. One of the most important strategies to minimize ASCVD risk involves treating dyslipidemia in both primary and secondary prevention settings [2]. During the last decade, a novel hypolipidemic drug category has emerged, targeting proprotein convertase subtilisin/kexin type 9 (PCSK9) [3,4]. PCSK9 is primarily synthesized by the liver and promotes low-density lipoprotein (LDL) receptor degradation, leading to increased circulating LDL-cholesterol (LDL-C) levels [5]. Therefore, inhibition of PCSK9 can lower LDL-C via promoting the recycling of the LDL receptors in the hepatocytes thus increasing LDL particle catabolism. Two PCSK9 monoclonal antibodies (PCSK9-mAb), i.e., alirocumab and evolocumab, are currently being used typically as add-ons to maximally tolerated statins with or without ezetimibe in hyperlipidemic patients at a high or very high CV risk [6,7]. These PCSK9-mAb have been shown to reduce LDL-C by 50–65% on top of statins (±ezetimibe), as well as lipoprotein a (Lp(a)) by a mean of 25–30% [8] and, most importantly, also major adverse cardiovascular events (MACE) with an overall excellent tolerability and safety profile [9,10]. Interestingly, this PCSK9-mAb-induced Lp(a) lowering was also associated with a significant reduction in CV outcomes [11].

Different pharmaceutical methods of PCSK9 inhibition are being developed, i.e., an anti-PCSK9 vaccine, for which human studies have just started [12], and nucleic acid-based therapies that inhibit or “silence” the expression of PCSK9 [9]. These therapeutics include small interfering RNAs (siRNA), N-acetylgalactosamine (GalNAc)-conjugated antisense oligonucleotides (ASO) and gene-editing options (e.g., clustered regularly interspaced short palindromic repeat (CRISPR) tools or meganucleases delivered by adeno-associated virus vectors) [13]. Among such agents, inclisiran, the first-in-class siRNA against PCSK9, has been recently approved by both the US Food and Drug Administration (FDA) and the European Medicines Agency (EMA), along with diet and maximally tolerated statin treatment for adults with heterozygous familial hypercholesterolemia (HeFH) or ASCVD who require additional LDL-C lowering [14,15].

Inclisiran can lower circulating LDL-C levels by halting PCSK9 gene translation intracellularly, thus leading to a decreased PCSK9 generation within the hepatocytes and subsequently to an increased number of LDL receptors [16]. Inclisiran therapy exerts certain advantages compared with the other LDL-C-reducing medications (i.e., statins, ezetimibe and PCSK9-mAb) since it offers a prolonged and significant LDL-C-lowering effect (although being administered only twice per year) with a very good safety profile so far. It is thus intuitive to expect that the infrequent dosing of inclisiran may represent an attractive option to overcome non-adherence to hypolipidemic therapy, which is a major cause of not achieving LDL-C targets with existing therapies. Furthermore, healthcare professionals can administer inclisiran during the bi-annual follow-up visits of their patients, thus minimizing the risk of both poor adherence and an improper drug administration technique by the patient. Therefore, the patient can be under tight control by his/her physician [17]. Overall, inclisiran provides a novel promising way to handle the huge burden of dyslipidemia and ASCVD. However, inclisiran’s long-term safety and effects on CV morbidity and mortality have not been established yet. Furthermore, the prolonged mechanism of action of inclisiran may affect the patients’ overall perception/acceptability of the drug if adverse effects were experienced [17].

The present narrative review discusses the effects of inclisiran on LDL-C and Lp(a) levels, with an emphasis on the **ORION/VICTORION** clinical program that has been designed to investigate the impact of inclisiran on atherogenic lipoproteins and MACE.

## 2. Summary of ORION/VICTORION Clinical Trials

The **ORION-1** trial was a phase 2 study that included 501 patients with ASCVD (*n* = 347, 69.3%) or ASCVD risk equivalents (*n* = 28 (6%) had FH, *n* = 118 (10.8%) had T2DM) [18]. Overall, 73% of participants were on statin therapy (*n* = 365) and 31% on ezetimibe (*n* = 151).

The **ORION-2** trial was a pilot, phase 3, open-label, single-arm, multicenter study conducted in four patients with homozygous familial hypercholesterolemia (HoFH) on maximally tolerated, high-intensity statins and ezetimibe [19]. Inclisiran sodium 300 mg was subcutaneously administered on day 1 in all four patients; three patients also received a second inclisiran injection on day 90, whereas the fourth patient received only one injection on day 1 since PCSK9 levels remained suppressed by >70% on days 60 and 90.

The **ORION-1** study was extended (up to 4 years) with 290 inclisiran-treated patients receiving only inclisiran (300 mg every 180 days) until the end of the follow-up (inclisiran-only arm) and 92 patients, initially receiving a placebo in the **ORION-1** study, followed by evolocumab 140 mg every 14 days up to day 360 and then transitioning to inclisiran (300 mg every 180 days) up to 4 years (switching arm) [20]. This phase 2, open-label extension trial was named **ORION-3**.

The most clinically relevant of the **ORION** program is the **ORION-4** CV outcome trial (CVOT). **ORION-4** (NCT03705234) is still recruiting and aims at evaluating the 5-year occurrence of major coronary adverse events in approximately 15,000 participants aged ≥55 years with pre-existing ASCVD randomized to either inclisiran sodium 300 mg (every 180 days) or matching placebo [21]. The primary endpoint of this phase 3 trial includes coronary heart disease (CHD) death, myocardial infarction (MI), fatal or non-fatal ischemic stroke and an urgent coronary revascularization procedure, whereas the composites of CHD death, MI or CV death represent secondary outcomes [21]. The estimated completion date for this study is July 2026.

In the **ORION-5** trial, a phase 3, two-part multicenter study (i.e., double-blind placebo-controlled for 6 months, followed by an 18-month open-label extension where all patients were treated with inclisiran), the efficacy of inclisiran was evaluated among 56 HoFH patients [22]. The results have been very recently posted (accessed on 24 March 2023, https://www.clinicaltrials.gov/ct2/show/results/NCT03851705?cond=NCT03851705&draw=2&rank=1), and thus we need to wait for the full publication to comment on them.

The **ORION-6** trial was a single-center, open-label, phase 1 parallel-group study involving patients with mild (*n* = 10) or moderate (*n* = 6) hepatic impairment (HI), as well as participants with normal hepatic function (*n* = 12) [23]. All participants received a single subcutaneous dose of inclisiran (300 mg).

In phase 1, open-label **ORION-7** trial, the effect of renal impairment (RI) on the pharmacokinetics, pharmacodynamics, safety and tolerability of a subcutaneously administered single dose of inclisiran sodium 300 mg was evaluated [24]. Among a total of 31 participants, 8 had normal renal function (defined as creatinine clearance (CrCl) ≥90 mL/min), 8 had mild RI (i.e., CrCl 60–89 mL/min), 8 had moderate RI (i.e., CrCl 30–59 mL/min), and 7 had severe RI (i.e., CrCl 15–29 mL/min).

The **ORION-8** trial (NCT03814187) is the open-label, long-term extension of the **ORION-9**, **ORION-10, ORION-11** and **ORION-5** studies [25]. This trial will evaluate the safety and efficacy of inclisiran in achieving lipid targets among 2991 patients with HeFH, HoFH, established ASCVD or ASCVD risk equivalents, followed up for 1080 days [25]. This trial is still ongoing.

Three pivotal studies in the ORION program were ORION 9, 10 and 11. In phase 3, double-blind, **ORION-9** trial, 482 HeFH patients were randomly assigned, in a 1:1 ratio, to receive inclisiran sodium (at a dose of 300 mg) or placebo on days 1, 90, 270 and 450 [26]. Patients were on a maximally tolerated statin (*n* = 436, 90.4%) (*n* = 356 on a high-intensity statin (73.8%)) and ezetimibe (*n* = 255, 52.9%).

The **ORION-10** trial was a randomized, double-blind, placebo-controlled, parallel-group, phase 3 study involving 1561 US patients with ASCVD (with LDL-C > 70 mg/dL) randomly assigned to receive, in a 1:1 ratio, either inclisiran (284 mg) or placebo, subcutaneously administered on days 1 and 90 and thereafter every 6 months until 540 days [27]. Patients were on a maximally tolerated statin (*n* = 1393, 89.2%) (*n* = 1062 on a high-intensity statin (68%)) and ezetimibe (*n* = 154, 9.9%).

In the randomized, double-blind, placebo-controlled, phase 3 **ORION-11** trial, 1617 patients from Europe and South Africa with ASCVD (*n* = 1414 with LDL > 70 mg/dL) or an ASCVD equivalent (*n* = 203; 132 with T2DM, 30 with HeFH and 41 with ≥20% 10-year Framingham Risk Score, with LDL-C > 100 mg/dL) were randomly assigned (1:1 ratio) to receive either inclisiran (284 mg) or placebo, administered subcutaneously on days 1 and 90 and thereafter every 6 months up to 540 days [27]. Patients were on a maximally tolerated statin (*n* = 1532, 94.7%) (*n* = 1271 on a high-intensity statin (78.6%)) and ezetimibe (*n* = 114, 7.1%).

Several other inclisiran clinical trials are planned such as **ORION-12** (phase 1, *n* = 48 healthy volunteers, outcome: ECG modifications) and **ORION-17** (phase 3, primary prevention trial, *n* = 40,000 participants) or ongoing, including **ORION-13, -14, -15, -16** and **-18**, as well as **VICTORION-INITIATE, VICTORION-INCEPTION** and **VICTORION-2 PREVENT** [28]. In brief, **ORION-13** (NCT04659863) is a phase 3 trial involving 15 adolescents (12–18 years) with HoFH who will receive either inclisiran or placebo for up to 720 days [29]; **ORION-14** (NCT04774003) is a phase 1 trial including 40 Chinese patients with increased LDL-C levels despite hypolipidemic treatment who will receive inclisiran 100 mg, inclisiran 300 mg or placebo for up to 90 days [30]; **ORION-15** is a phase 2 study involving 308 Japanese patients with ASCVD and elevated LDL-C levels on inclisiran 300, 200 or 100 mg vs. placebo for up to 180 days [28]; **ORION-16** (NCT04652726) is a phase 3 study with 150 adolescents (12–18 years) with HeFH on inclisiran 300 mg or placebo for up to 720 days [31]; and **ORION-18** (NCT04765657) is a phase 3 trial including 320 Asians with ASCVD or ASCVD risk equivalents who will receive either inclisiran 300 mg or placebo for 360 days (+extension to 3 years) [32].

The **VICTORION** trials will evaluate the efficacy of inclisiran in patients with CVD. In particular, **VICTORION-INITIATE** (NCT04929249) is a phase 3 study including 444 patients with ASCVD and LDL-C > 70 mg/dL on a maximally tolerated statin who will receive inclisiran 300 mg + usual care vs. usual care for up to 330 days [33], whereas **VICTORION-INCEPTION** (NCT04873934) is a phase 3 study involving 384 patients with recent (within 5 weeks) acute coronary syndrome (ACS) who will be treated with inclisiran 300 mg + usual care vs. usual care for up to 360 days [34]. **VICTORION-2 PREVENT** (NCT05030428) is the most important of the VICTORION clinical trial program since it is a CVOT—a phase 3 clinical trial which will include 15,000 patients with established CVD that will receive inclisiran 300 mg vs. placebo and will be followed up for 6 years [35]. This study will evaluate not only the effects of inclisiran on lipid parameters but also on the occurrence of MACE (i.e., nonfatal MI, nonfatal stroke, CV death, urgent coronary revascularization), as well as all-cause death [35]. The study is still recruiting, and the estimated completion date is October 2027.

Other currently recruiting clinical trials are **VICTORION-REAL** (NCT05399992), which will evaluate the effectiveness and adherence of inclisiran in ASCVD patients [36], **VICTORION-DIFFERENCE** (NCT05192941) [37], which will examine the efficacy, tolerability, safety and quality of life (in relation to drug-related adverse effects) of inclisiran in patients with hypercholesterolemia treated with rosuvastatin, **VICTORION-IMPLEMENT** (NCT05362903) [38], which is a non-interventional implementation real-world study, **VICTORION-PLAQUE** (NCT05360446) [39], which will assess the impact of inclisiran therapy on atherosclerotic plaque progression in patients with non-obstructive CHD without previous CV events, and **VICTORION-SPIRIT** (NCT04807400), a phase 3 clinical study that will evaluate the implementation preference and utility of inclisiran administration in patients with ASCVD or ASCVD risk equivalents in a regional primary care setting, i.e., the National Health System (NHS) [40].

Table 1 summarizes the patient populations of the **ORION** and **VICTORION** trials.

**Figure 1:**
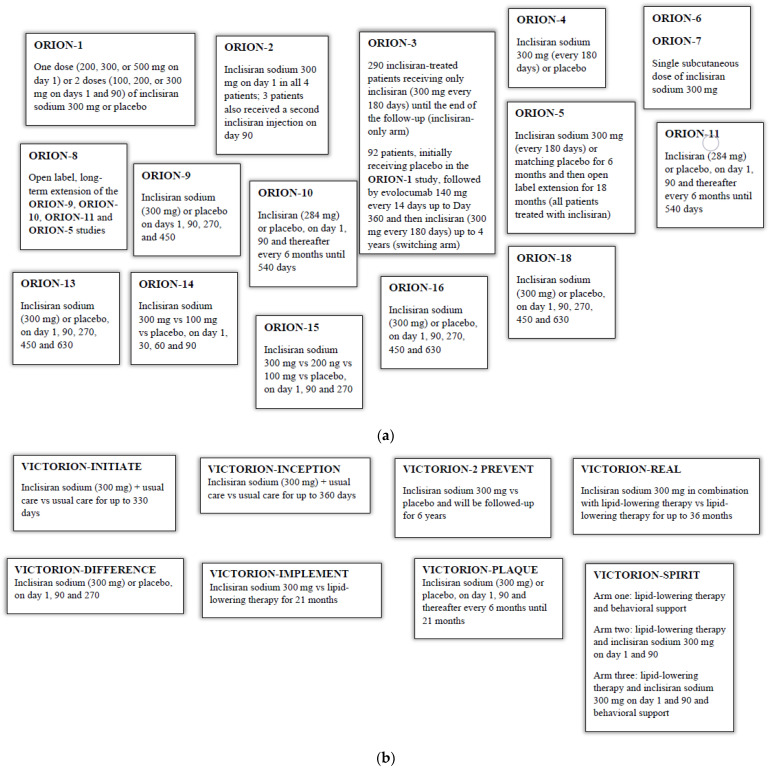
(**a**) Inclisiran administration in the **ORION** trials; (**b**) Inclisiran administration in the **VICTORION** trials.

Figure 1a,b summarize the different drug treatments in the above-mentioned clinical trials.

## 3. Effects of Inclisiran on LDL-C

In the **ORION-1** trial, baseline mean (±SD) LDL-C was 128 (±50) mg/dL [18]. The time-averaged decrease in LDL-C levels over 1 year ranged from 29.5 to 38.7% after a single dose (*p* < 0.001 between groups) and from 29.9 to 46.4% in those who received two doses (*p* < 0.001 between groups). The highest mean LDL-C reduction over 1 year and the greatest proportion of responders at day 360 were observed in those receiving a two-dose 300 mg regimen [18].

In the **ORION-2** trial, among four HoFH patients, LDL-C was durably lowered in three patients by −11.7% to −33.1% at day 90 and by −17.5% to −37.0% at day 180 [19]. However, in one patient, no reduction in LDL-C levels was observed despite achieving a durable PCSK9 decrease throughout the study. It should be noted that this participant was also a poor responder to both alirocumab and evolocumab [19].

In the inclisiran-only arm of the **ORION-3** trial, LDL-C levels were decreased by 47.5% (95% CI 50.7–44.3; *p* < 0.0001) at day 210 (i.e., approximately 570 days after the first inclisiran administration in the **ORION-1** study). This reduction was sustained over 1440 days (−46.7%, 95% CI −50.7 to −42.8) [20]. The 4-year averaged mean LDL-C reduction was 44.2% (95% CI: 47.1–41.4) in the inclisiran-only arm (i.e., after nine inclisiran injections); the corresponding value for the switching arm was −45.3% (95% CI −49.7 to −40.9), depicting exposure to inclisiran from year 2 to year 4 (i.e., 7 injections) [20]. Therefore, similar time-averaged LDL-C reductions were achieved in both study arms, suggesting that previous exposure/therapy with a monoclonal antibody against PCSK9 did not affect the efficacy of inclisiran. Furthermore, the transition from evolocumab to inclisiran was either staged or concurrent; no meaningful differences in terms of safety or efficacy were observed between these two modalities of transition to inclisiran, thus highlighting the absence of any special consideration when switching the therapy from evolocumab to inclisiran [20]. No safety issues were raised; 14% of patients in each study arm reported injection-site reactions, whereas treatment-emergent serious adverse events possibly linked to the study drug were recorded for only 1% of patients in both study groups [20].

In the **ORION-6** trial, the pharmacokinetic exposure of inclisiran was increased by 1.24 and 2.03-fold in patients with mild and moderate HI compared with those with normal liver function, respectively [23]. Mean LDL-C change from baseline to day 60 was −51.9% and −53.2% in the normal hepatic function and mild HI groups, respectively, compared with −39.7% in patients with moderate HI [23]. The corresponding mean absolute LDL-C changes were 68.5 mg/dL (mean baseline level: 132.9 mg/dL), 53.2 mg/dL (97.4 mg/dL) and 79.2 mg/dL (160.0 mg/dL), respectively. Inclisiran was proven generally safe and well-tolerated in all participant groups, with no dose adjustment needed for those with HI [23]. However, larger and longer-duration clinical trials are needed to establish the efficacy and safety of inclisiran in the presence of HI.

In the **ORION-7** trial, mean (±SD) LDL-C decreases at day 60 were similar for participants with normal renal function (57.6 ± 10.7%) and mild (35.1 ± 13.5%), moderate (53.1 ± 21.3%) or severe RI (49.2 ± 26.6%) (*p* = 0.17 for participants with normal renal function vs. those with any RI) [24]. These reductions persisted up to the end of the study at day 180, thus highlighting that dose adjustments of inclisiran are not required in these patients [24]. The tolerability and safety profile of inclisiran were similar irrespective of renal function.

In the **ORION-9** trial, baseline mean (±SD) LDL-C values and Lp(a) were 151 (±50) mg/dL and 54 (IQR 20–185) nmol/L in the inclisiran group (*n* = 242 patients) and 155 (±58) mg/dL and 57 (IQR 22–180) nmol/L in the placebo group (*n* = 240 patients), respectively. The first primary endpoint was the % change in LDL-C levels from baseline to day 510 which was a reduction of 39.7% (95% CI −43.7 to −35.7) in the inclisiran group and an increase of 8.2% (95% CI 4.3 to 12.2) in the placebo group (between-group difference: −47.9%, 95% CI −53.5 to −42.3; *p* < 0.001) [26]. The time-averaged % change in LDL-C between days 90 and 540 was a reduction of 38.1% (95% CI −41.1 to −35.1) in the inclisiran group and an increase of 6.2% (95% CI 3.3 to 9.2) in the placebo group (between-group difference: −44.3%, 95% CI −48.5 to −40.1; *p* < 0.001) [26]. Furthermore, the mean absolute change in LDL-C levels from baseline to day 510 was −59.0 mg/dL (95% CI −64.8 to −53.2) (1.5 mmol/L, 95% CI −1.7 to −1.4) in the inclisiran group and +9.9 mg/dL (95% CI 4.1 to 15.8) (0.3 mmol/L, 95% CI 0.1 to 0.4) in the placebo group (between-group difference: −68.9 mg/dL, 95% CI −77.1 to −60.7 (1.8 mmol/L, 95% CI −2.0 to −1.6); *p* < 0.001) [26]. The time-averaged difference in LDL-C values between day 90 and day 540 was −56.9 mg/dL (−1.5 mmol/L) and 5.8 mg/dL (0.1 mmol/L) in the inclisiran and placebo groups, respectively (between-group difference: −62.6 mg/dL (−1.6 mmol/L); *p* < 0.001) [26].

The **ORION-10** trial, mean ± SD baseline LDL-C levels were 105 ± 38.3 mg/dL (2.7 ± 0.9 mmol/L) and 105 ± 39 mg/dL (2.7 ± 1.0 mmol/L) in the inclisiran group (*n* = 781) and the placebo group (*n* = 780), respectively. The % change in LDL-C levels at day 510 was −51.3% in the inclisiran group and +1.0% in the placebo group (between-group difference: −52.3%, 95% −55.7 to −48.8; *p* < 0.001) [27]. The corresponding absolute changes were −56.2 mg/dL (−1.45 mmol/L) in the inclisiran group and −2.1 mg/dL (−0.05 mmol/L) in the placebo (between-group difference: −54.1 mg/dL (−1.40 mmol/L), 95% CI −57.4 to −50.9 mg/dL (−1.48 to −1.32 mmol/L]; *p* < 0.001). The time-adjusted % change in LDL-C from day 90 to day 540 compared with baseline was −51.3% in the inclisiran group and +2.5% in the placebo group (between-group difference: −53.8%, 95% CI −56.2 to −51.3; *p* < 0.001) [27]. The corresponding absolute changes were −53.7 mg/dL (−1.39 mmol/L) in the inclisiran group and −0.4 mg/dL (−0.01 mmol/L) in the placebo group (between-group difference: −53.3 mg/dL (−1.38 mmol/L), 95% CI −55.8 to −50.8 mg/dL (−1.44 to −1.31 mmol/L); *p* < 0.001) [27].

In the **ORION-11** trial, mean (±SD) baseline LDL-C values were 107 (±42) and 104 (±36) mg/dL in the inclisiran and placebo groups, respectively. The % changes in LDL-C values at day 510 were −45.8% in the inclisiran group and +4.0% in the placebo group (between-group difference: −49.9%, 95% CI −53.1 to −46.6; *p* < 0.001) [27]. The corresponding absolute changes were −50.9 mg/dL (−1.32 mmol/L) in the inclisiran group and +1.0 mg/dL (0.03 mmol/L) in the placebo group (between-group difference: −51.9 mg/dL (−1.34 mmol/L), 95% CI −55.0 to −48.7 mg/dL (−1.42 to −1.26 mmol/L); *p* < 0.001). The time-adjusted % changes in LDL-C levels from day 90 to day 540 were −45.8% in the inclisiran group and +3.4% in the placebo group (between-group difference: −49.2%, 95% CI −51.6 to −46.8; *p* < 0.001) [27]. The corresponding absolute changes were −48.6 mg/dL (−1.26 mmol/L) in the inclisiran group and +0.3 mg/dL (0.01 mmol/L) in the placebo group (between-group difference: −48.9 mg/dL (−1.26 mmol/L), 95% CI −51.4 to −46.5 mg/dL (−1.33 to −1.20 mmol/L); *p* < 0.001) [27].

In a post hoc analysis of the **ORION-9, -10 and -11** trials, twice-yearly inclisiran therapy was well-tolerated and effective in LDL-C reduction in both patients with (*n* = 470) and without polyvascular disease (PVD) (*n* = 2984) [41]. In brief, mean (95% CI) placebo-corrected LDL-C % change from baseline to day 510 was −48.9% (−55.6 to −42.2) in patients with PVD and −51.5% (−53.9 to −49.1) in patients without [41].

It should be noted that inclisiran therapy was generally very well-tolerated in all clinical trials causing only a significant increase in injection-site reaction by about 8.2% (in the **ORION-9-11** trials) [26,27]. Furthermore, both laboratory-defined and system-organ adverse events were similar in the inclisiran and placebo groups. As mentioned above, only injection-site reactions were more frequent in inclisiran-treated patients compared with placebo; such reactions were generally mild, and none were severe or persistent [26,27].

## 4. Effects of Inclisiran on Lp(a)

In the **ORION-1** trial, involving 501 participants (69% with ASCVD; 73% on statins, mean LDL-C 128 mg/dL), median Lp(a) levels were decreased from baseline at day 150 by −15% to −19% in the single-dose inclisiran groups and by −19% to −25% in the two-dose inclisiran groups [42]. The corresponding ranges at day 180 were −14% to −18% in the single-dose groups and −15% to −26% in the two-dose groups. However, none of the observed Lp(a) reductions reached statistical significance, potentially due to substantial interindividual variability in these changes [42]. Despite this variation in the magnitude of the inclisiran effect on Lp(a), almost 90% of the participants in the 300 mg two-dose group had a 26% median Lp(a) decrease at day 180 which is quite similar to the impact typically found in trials with the other PCSK9i [41]. Of note, median baseline Lp(a) values ranged from 25.3 to 47.0 nmol/L.

In a sub-analysis of the **ORION-1** trial (*n* = 501 participants), inclisiran was associated with lower Lp(a) levels regardless of the presence (*n* = 67) or absence (*n* = 415) of T2DM. It was not reported whether this decrease was significant [43].

In the **ORION-3** trial, the open-label extension of **ORION-1**, at day 1440, Lp(a) levels were reduced by 6.3% in the inclisiran-only arm and by 14.3% in the switching arm (*p* values were not reported) [20].

The **ORION-9** trial included 482 HeFH patients who received either inclisiran sodium (at a dose of 300 mg) or placebo (in a 1:1 ratio) [26]. Median (IQR) baseline Lp(a) values were 57 (22–180) nmol/L in the inclisiran group (*n* = 242 patients) and 54 (20–185) nmol/L in the placebo group (*n* = 240 patients), respectively. At day 540, Lp(a) levels were decreased by −13.5% in the inclisiran group, whereas they were increased by 3.7% in the placebo group (placebo-adjusted difference: −17.2%). Significance levels were not provided [26].

In the **ORION-10** trial (*n* = 1561 US patients with ASCVD), baseline median (IQR) Lp(a) values were 57 (18–181) nmol/L in the inclisiran group and 56 (20–189) nmol/L in the placebo group [26]. At day 540, median Lp(a) levels were lowered by 21.9% in the inclisiran group, whereas they increased by 3.7% in the placebo group (placebo-adjusted change: −25.6%) [27]. However, if this decrease was significant or not remains unclear. *p* values were not shown as these did not take into account the multiplicity of testing. Similarly, in the **ORION-11** trial (*n* = 1617 patients from Europe and South Africa with ASCVD or an ASCVD equivalent, i.e., T2DM, FH or a ≥20% Framingham Risk Score), at day 540, median Lp(a) values were decreased by 18.6% in the inclisiran group, whereas they remained the same in the placebo group (placebo-adjusted change: −18.6%) [27]. Again, for the reasons mentioned above, *p* values could not be calculated, so the significance of this reduction remains unclear. Baseline median (IQR) Lp(a) was 42 (18–178) and 35 (18–181) nmol/L in the inclisiran and placebo groups, respectively.

There was, however, one study that showed a significant decrease in Lp(a) with inclisiran treatment. A pre-specified analysis of the **ORION-11** trial included 203 individuals at risk of CV events and baseline LDL-C ≥2.6 mmol/L (100 mg/dL), despite maximally tolerated statins [44]. This “primary prevention cohort” (referred to as “risk equivalent” in the study protocol) involved patients with FH, T2DM or with a 10-year risk of ≥20% of a CV event (assessed by the Framingham Risk Score). At baseline, median (IQR) Lp(a) values were 34 (14, 142), 40 (17, 148) and 27 (14, 138) nmol/L for the total population (*n* = 203), the inclisiran group (*n* = 98) and the placebo group (*n* = 105) [44]. The placebo-corrected percentage decrease in Lp(a) levels from baseline to day 540 was 28.5%; time-adjusted absolute change (mean, 95% CI) was −12.5 (−17.1, −8.0) nmol/L for the inclisiran group and +5.5 (1.2, 9.9) nmol/L for the placebo group; LS difference was −18.1 (−24.3, −11.8) nmol/L (*p* < 0.0001) [44]. The unusual 16.8% increase in Lp(a) levels observed in the placebo group may have contributed to the difference between the groups being significant.

Larger outcome trials with inclisiran will conclusively answer the question of whether inclisiran decreases Lp(a) levels as the PCSK9 antibodies do.

## 5. Effects of Inclisiran on Other Lipoproteins and Apolipoproteins

In a phase 1 trial, involving healthy volunteers, inclisiran significantly decreased not only LDL-C levels (up to a least-squares mean reduction of 50.6% in the single-dose groups and 59.7% in the multiple-dose groups) but also total cholesterol (TC), non-high-density lipoprotein cholesterol (non-HDL-C) and apolipoprotein B (apoB) in both single-dose groups (*n* = 24; from baseline to day 84) and multiple-dose regimes (*n* = 45; from baseline to day 180) [45]. Of note, among single-dose groups, the greatest reductions in TC, apoB and non-HDL-C were observed in the 300 mg inclisiran group (i.e., −30.9, −47.1 and −48.9%, respectively). Similar results were seen among the multiple-dose groups with corresponding values −40.4, −56.9 and −52.4%, respectively) [45].

In the **ORION-1** trial, significant reductions in TC, non-HDL-C and apoB were observed at day 180 in all single- and two-dose inclisiran groups [42]. In brief, TC percent changes from baseline ranged from −18 to −27% in the single-dose groups and from −22 to −33% in the two-dose groups (*p* < 0.001 for all comparisons with placebo), whereas for non-HDL-C, the corresponding values were −25 to −37% and −32 to −46%, respectively, and for apoB, they were from −23 to −33% and −28 to −41%, respectively (*p* < 0.001 for all comparisons with placebo) [42]. Therefore, the observed decreases in TC, non-HDL-C and apoB following inclisiran administration were dose-dependent. Interestingly, at day 180, the vast majority of patients in the 300 mg two-dose inclisiran group achieved apoB goals: 78.0% attained <80 mg/dL and 89.8% <100 mg/dL; the corresponding value for non-HDL-C was 67.8% for <100 mg/dL and 83.1% for <130 mg/dL [42]. Even among patients receiving a single dose of 300 mg inclisiran, 73.3% achieved apoB <80 mg/dL and 83.3% <100 mg/dL at day 180, whereas 61.7% and 78.3% had non-HDL-C <100 and <130 mg/dL, respectively [42].

It should also be noted that, in contrast to the homogeneous decreases in TC, non-HDL-C and apoB, there was considerable temporal and interindividual variability in very low density lipoprotein cholesterol (VLDL-C) reductions seen in the **ORION-1** trial [42]. Furthermore, VLDL-C was not reduced at day 180 in almost 25% of the participants in the 300 mg two-dose group. Substantial interindividual variation was also observed in relation to changes in triglyceride (TG) levels [42]. At day 180, significant TG decreases from baseline were observed only in the 300 and 500 mg single-dose groups (−13% (*p* < 0.01 vs. placebo) and −12% (*p* < 0.05 vs. placebo), respectively) and in the 300 mg two-dose group (−14%; *p* < 0.05). In the same trial (i.e., **ORION-1**), HDL-C and apoA1 were modestly increased [42]. At day 180, HDL-C was significantly raised only in the 300 mg single-dose group (9% from baseline; *p* < 0.05 vs. placebo) and in all two-dose groups (8% (*p* < 0.05), 10% (*p* < 0.001) and 9% (*p* < 0.01) for the 100, 200 and 300 mg, respectively) [42]. The percentage change from baseline for apoA1 at day 180 ranged from 3 to 4% in the single-dose groups and from 6 to 9% in the two-dose groups (*p* < 0.05 versus placebo only in the 200 and 300 mg groups) [42].

In the **ORION-3** trial, the % change from baseline to day 1440 was −28.6 (−31.6 to −25.7) for TC, −40.4 (−44.2 to −36.6) for non-HDL-C, +9.1 (6.3 to 11.9) for HDL-C, −33.7 (−37.2 to −30.2) for apoB and −12.0 (−17.8 to −5.6) for TG in the inclisiran-only arm [20]. The corresponding values for the switching arm were −27.4 (−31.8 to −23.0) for TC, −39.8 (−45.4 to −34.2) for non-HDL-C, +11.0 (6.0 to 15.9) for HDL-C, −34.9 (−39.5 to −30.2) for apoB and −4.2 (−16.9 to +3.5) for TG. *p* values were not provided [20].

In the **ORION-9** trial, at day 510, inclisiran was reported to decrease TC (−32.9% placebo adjusted), non-HDL-C (−43.6% placebo adjusted), apoB (−36.9% placebo adjusted) and TG (−11.8% placebo adjusted) (*p* values not provided) [26]. HDL-C was modestly increased (+2.6% placebo adjusted) [28]. Similar improvements in lipid parameters were observed with inclisiran therapy in the **ORION-10** and **ORION-11** trials [27]. In brief, in the **ORION-10** trial, placebo-adjusted changes at day 510 were −33.1% for TC (*p* < 0.001), −47.4% for non-HDL-C (*p* < 0.001), −43.1% for apoB (*p* < 0.001), −12.6% for TG (*p* not provided) and +5.1% for HDL-C (*p* not provided) [27]. The corresponding values in the **ORION-11** trial were −29.8% for TC (*p* < 0.001), −43.3% for non-HDL-C (*p* < 0.001), −38.9% for apoB (*p* < 0.001), −7.0% for TG (*p* not provided) and +6.1% for HDL-C (*p* not provided) [27].

In the pre-specified analysis of the **ORION-11** trial, involving the “primary prevention cohort” (referred to as “risk equivalent” in the study protocol), the mean placebo-corrected percentage changes from baseline to day 510 were −39.5% for non-HDL-C and −35.8% for apoB (*p* < 0.0001 for both comparisons) [44]. Similar results were found for TC and in the secondary prevention cohort [44]. Interestingly, at day 510, higher proportions of patients on inclisiran (vs. placebo) achieved apoB levels <100 mg/dL and <80 mg/dL: 81.2 vs. 38.3% (OR 6.9, 95% CI 3.5–13.8) and 61.2 vs. 7.4% (OR 19.6, 95% CI 8.1–47.5), respectively [44]. The corresponding values for attaining non-HDL-C levels <3.4 mmol/L (130 mg/dL) and <2.6 mmol/L (100 mg/dL) were 76.5 vs. 27.4% (OR 8.6, 95% CI 4.4–16.9) and 49.4 vs. 4.2% (OR 22.2, 95% CI 7.5–65.9), respectively [44]. Similar findings were observed in the secondary prevention group of the **ORION-11** trial [44].

Based on these data, inclisiran can beneficially affect TC, non-HDL-C and apoB levels, thus increasing the proportion of patients achieving the goals for these lipid variables. The implications of these benefits in clinical practice remain to be established.

Table 2 summarizes the effects of inclisiran on lipids and lipoproteins reported by the above-mentioned trials.

## 6. Conclusions

Inclisiran exerts a prolonged and significant LDL-C-lowering effect based on its unique mechanism of action. Some of the **ORION** trials have been completed confirming that inclisiran effectively reduced LDL-C in patients with elevated LDL-C, despite maximally tolerated statin therapy (±ezetimibe), in different clinical settings (i.e., patients with HoFH, HeFH, ASCVD or ASCVD risk equivalents, as well as in CKD and PVD patients) [46,47]. Furthermore, novel findings were reported showing inclisiran-induced decreases in apoB, TC, non-HDL-C and Lp(a), as well as modest increases in HDL-C levels. Very recently, long-term exposure to inclisiran (up to 5 years), as well as switching from evolocumab to inclisiran, was reported to be both safe and efficient in achieving and sustaining LDL-C reductions in the **ORION-3** study [20].

We still need data on the early efficacy of inclisiran (when we might observe the significant LDL-C reduction after injection), in patients with early ACS (within the first 3 months), on atheroma plaque volume as well as in other patient populations such as those with statin intolerance, peripheral artery disease and the elderly. Further studies/real-world data will also answer the questions on LDL-C reduction efficacy in comparison to PCSK9 inhibitors. Overall, inclisiran was well-tolerated with a similar safety profile with placebo, except for mild and transient injection-site reactions. Regarding reducing CV events, a recent patient-level analysis of **ORION-9**, -**10** and **-11** found that inclisiran significantly reduced composite MACE but not fatal or non-fatal MIs or fatal and non-fatal stroke [48]. The long-term safety and efficacy of inclisiran will be unequivocally reflected in the eagerly awaited findings of the larger and longer CV endpoint trials **ORION-4** and **VICTORION-2 PREVENT**, which will also determine the clinical relevance of this promising new treatment.

## Figures and Tables

**Table 1:** Patient populations in the **ORION** and **VICTORION** trials.

Completed Clinical Trials	
**ORION-1**	501 patients with ASCVD (*n* = 347, 69.3%) or ASCVD risk equivalents (*n* = 28 (6%) had FH, *n* = 118 (10.8%) had T2DM) 73% of participants were on statin therapy (*n* = 365) and 31% on ezetimibe (*n* = 151)
**ORION-2**	4 patients with HoFH on maximally tolerated, high-intensity statins and ezetimibe
**ORION-3**	**ORION-1** study extension (up to 4 years) 290 inclisiran-treated patients receiving only inclisiran (300 mg every 180 days) until the end of the follow-up (inclisiran-only arm) and 92 patients initially receiving placebo in the **ORION-1** study, followed by evolocumab 140 mg every 14 days up to day 360 and then transitioning to inclisiran (300 mg every 180 days) up to 4 years (switching arm)
**ORION-5**	56 HoFH patients
**ORION-6**	Patients with mild (*n* = 10) or moderate (*n* = 6) hepatic impairment, as well as participants with normal hepatic function (*n* = 12)
**ORION-7**	31 participants: 8 had normal renal function (defined as creatinine clearance (CrCl) ≥ 90 mL/min), 8 had mild renal impairment (i.e., CrCl 60–89 mL/min), 8 had moderate renal impairment (i.e., CrCl 30–59 mL/min), and 7 had severe renal impairment (i.e., CrCl 15–29 mL/min)
**ORION-9**	482 HeFH patients on maximally tolerated statin (*n* = 436, 90.4%) (*n* = 356 on high-intensity statin (73.8%)) and ezetimibe (*n* = 255, 52.9%)
**ORION-10**	1561 US patients with ASCVD (with LDL-C > 70 mg/dL) on maximally tolerated statin (*n* = 1393, 89.2%) (*n* = 1062 on high-intensity statin (68%)) and ezetimibe (*n* = 154, 9.9%)
**ORION-11**	1617 patients from Europe and South Africa with ASCVD (*n* = 1414 with LDL > 70 mg/dL) or an ASCVD equivalent (*n* = 203; 132 with T2DM, 30 with HeFH and 41 with ≥20% 10-year Framingham Risk Score, with LDL-C > 100 mg/dL) on maximally tolerated statin (*n* = 1532, 94.7%) (*n* = 1271 on high-intensity statin (78.6%)) and ezetimibe (*n* = 114, 7.1%)
**Ongoing clinical trials**	
**ORION-4**	15,000 participants aged ≥55 years or older with pre-existing ASCVD
**ORION-8**	Open-label, long-term extension of the **ORION-9, ORION-10, ORION-11** and **ORION-5** studies
**ORION-12**	48 healthy volunteers
**ORION-13**	15 adolescents (12–18 years) with HoFH
**ORION-14**	40 Chinese patients with increased LDL-C levels despite hypolipidemic treatment
**ORION-15**	308 Japanese patients with ASCVD and elevated LDL-C levels
**ORION-16**	150 adolescents (12–18 years) with HeFH
**ORION-18**	320 Asians with ASCVD or ASCVD risk equivalents
**VICTORION-INITIATE**	444 patients with ASCVD and LDL-C > 70 mg/dL on maximally tolerated statin
**VICTORION- INCEPTION**	384 patients with recent (within 5 weeks) ACS
**VICTORION- 2 PREVENT**	15,000 patients with established CVD
**VICTORION- REAL**	ASCVD patients
**VICTORION- DIFFERENCE**	Patients with hypercholesterolemia treated with rosuvastatin at very-high or high CV risk
**VICTORION- IMPLEMENT**	Patients with hypercholesterolemia or ASCVD
**VICTORION- PLAQUE**	Patients with non-obstructive CHD without previous CV events
**VICTORION- SPIRIT**	Patients with ASCVD or ASCVD risk equivalents

ASCVD: atherosclerotic cardiovascular disease, CV: cardiovascular, FH: familial hypercholesterolemia, HoFH: homozygous familial hypercholesterolemia, HeFH: heterozygous familial hypercholesterolemia, T2DM: type 2 diabetes mellitus, CHD: coronary heart disease, ACS: acute coronary syndrome, LDL-C: low-density lipoprotein cholesterol.

**Table 2:** Effects of inclisiran on lipids and lipoproteins in the ORION clinical trials.

Clinical Trial	Effects on Lipids and Lipoproteins
**ORION-1** [20,44]	Time-averaged decrease in LDL-C over 1 year: 29.5 to 38.7% after a single dose (*p* < 0.001 between groups) and 29.9 to 46.4% after 2 doses (*p* < 0.001 between groups) Median Lp(a) level decreases at day 150: −15% to −19% in the single-dose inclisiran groups and −19% to −25% in the 2-dose inclisiran groups Median Lp(a) level decreases at day 180: −14% to −18% in the single-dose groups and −15% to −26% in the 2-dose groups Mean % changes in TC at day 180: −18 to −27% in the single-dose groups and −22 to −33% in the 2-dose groups (*p* < 0.001 for all comparisons with placebo) Mean % changes in non-HDL-C at day 180: −25 to −37% in the single-dose groups and −32 to −46% in the 2-dose groups (*p* < 0.001 for all comparisons with placebo) Mean % changes in apoB at day 180: −23 to −33% in the single-dose groups and −28 to −41% in the 2-dose groups (*p* < 0.001 for all comparisons with placebo) Mean % changes in TG at day 180: −13% in the 300 mg single-dose groups (*p* < 0.05 vs. placebo), −12% in the 500 mg single-dose groups (*p* < 0.05 vs. placebo) and −14% in the 300 mg 2-dose group (*p* < 0.05 vs. placebo) Mean % changes in HDL-C at day 180: +9% in the 300 mg single-dose groups (*p* < 0.05 vs. placebo), +8% in the 100 mg single-dose groups (*p* < 0.05 vs. placebo), +10% in the 200 mg 2-dose group (*p* < 0.001 vs. placebo) and +9% in the 300 mg 2-dose group (*p* < 0.01 vs. placebo)
**ORION-2** [21]	LDL-C reduction in 3 patients: at day 90: −11.7% to −33.1% at day 180: −17.5% to −37.0% In 1 patient: no reduction
**ORION-3** [22]	In the inclisiran-only arm: mean LDL-C decreases by 47.5% (95% CI 50.7–44.3; *p* < 0.0001) at day 210 and by 46.7% (95% CI 47.1–41.4) at day 1440 4-year averaged mean LDL-C reduction: 44.2% (95% CI: 47.1–41.4) (i.e., after 9 inclisiran injections) Median Lp(a) level decreases at day 1440: −6.3% in the inclisiran-only arm and −14.3% in the switching arm Mean % changes in TC at day 1440: −28.6% in the inclisiran-only arm and −27.4% in the switching arm Mean % changes in non-HDL-C at day 1440: −40.4% in the inclisiran-only arm and −39.8% in the switching arm Mean % changes in HDL-C at day 1440: +9.1% in the inclisiran-only arm and +11.0% in the switching arm Mean % changes in apoB at day 1440: −33.7% in the inclisiran-only arm and −34.9% in the switching arm Mean % changes in TG at day 1440: −12.0% in the inclisiran-only arm and −4.2% in the switching arm
**ORION-6** [25]	Mean LDL-C change at day 60: −51.9% in the normal hepatic function group −53.2% in the mild hepatic impairment group −39.7% in the moderate hepatic impairment group
**ORION-7** [26]	Mean LDL-C change at day 60: −57.6% in the normal renal function group −35.1% in the mild renal impairment group −53.1% in the moderate renal impairment group −49.2% in the severe renal impairment group
**ORION-9** [28]	Mean LDL-C change at day 510: −39.7% (95% CI −43.7 to −35.7) in the inclisiran group vs. +8.2% (95% CI 4.3 to 12.2) in the placebo group (between-group difference: −47.9%, 95% CI −53.5 to −42.3; *p* < 0.001) Time-averaged % LDL-C change between days 90 and 540: −38.1% (95% CI −41.1 to −35.1) in the inclisiran group vs. +6.2% (95% CI 3.3 to 9.2) in the placebo group (between-group difference: −44.3%, 95% CI −48.5 to −40.1; *p* < 0.001) Median Lp(a) level decreases at day 540: −13.5% in the inclisiran group vs. +3.7% in the placebo group (placebo-adjusted difference: −17.2%) Mean % changes in TC at day 510: −32.9% in the inclisiran group (placebo adjusted) Mean % changes in non-HDL-C at day 510: −43.6% in the inclisiran group (placebo adjusted) Mean % changes in HDL-C at day 510: +2.6% in the inclisiran group (placebo adjusted) Mean % changes in apoB at day 510: −36.9% in the inclisiran group (placebo adjusted) Mean % changes in TG at day 510: −11.8% in the inclisiran group (placebo adjusted)
**ORION-10** [29]	Mean LDL-C change at day 510: −51.3% in the inclisiran group vs. +1.0% in the placebo group (between-group difference: −52.3%, 95% CI −55.7 to −48.8; *p* < 0.001) Time-averaged % LDL-C change between days 90 and 540: −51.3% in the inclisiran group vs. +2.5% in the placebo group (between-group difference: −53.8%, 95% CI −56.2 to −51.3; *p* < 0.001) Median Lp(a) level decreases at day 540: −21.9% in the inclisiran group vs. +3.7% in the placebo group (placebo-adjusted difference: −25.6%) Mean % changes in TC at day 510: −33.1% in the inclisiran group (placebo adjusted) Mean % changes in non-HDL-C at day 510: −47.4% in the inclisiran group (placebo adjusted) Mean % changes in HDL-C at day 510: +5.1% in the inclisiran group (placebo adjusted) Mean % changes in apoB at day 510: −43.1% in the inclisiran group (placebo adjusted) Mean % changes in TG at day 510: −12.6% in the inclisiran group (placebo adjusted)
**ORION-11** [29]	Mean LDL-C change at day 510: −45.8% in the inclisiran group vs. +4.0% in the placebo group (between-group difference: −49.9%, 95% CI −53.1 to −46.6; *p* < 0.001) Time-averaged % LDL-C change between days 90 and 540: −45.8% in the inclisiran group vs. +3.4% in the placebo group (between-group difference: −49.2%, 95% CI −51.6 to −46.8; *p* < 0.001) Median Lp(a) level decreases at day 540: −18.6% in the inclisiran group vs. +0% in the placebo group (placebo-adjusted difference: −18.6%) Mean % changes in TC at day 510: −29.8% in the inclisiran group (placebo adjusted) Mean % changes in non-HDL-C at day 510: −43.3% in the inclisiran group (placebo adjusted) Mean % changes in HDL-C at day 510: +6.1% in the inclisiran group (placebo adjusted) Mean % changes in apoB at day 510: −38.9% in the inclisiran group (placebo adjusted) Mean % changes in TG at day 510: −7.0% in the inclisiran group (placebo adjusted)

LDL-C: low-density lipoprotein cholesterol; Lp(a): lipoprotein a; HDL-C: high-density lipoprotein cholesterol; TC: total cholesterol; TG: triglycerides; apoB: apolipoprotein B.

## Data Availability

Data is contained within the article.
